# Urine Levels of Defensin α1 Reflect Kidney Injury in Leptospirosis Patients

**DOI:** 10.3390/ijms17101637

**Published:** 2016-09-27

**Authors:** Haorile Chagan-Yasutan, Yue Chen, Talitha Lea Lacuesta, Prisca Susan A. Leano, Hiroko Iwasaki, Firmanto Hanan, Delsi Taurustiati, Yasukazu Ohmoto, Yugo Ashino, Hiroki Saitoh, Hideyasu Kiyomoto, Yasuhiko Suzuki, Freda O. Elizabeth Telan, Toshio Hattori

**Affiliations:** 1 Disaster-Related Infectious Disease, International Research Institute of Disaster Science, Tohoku University, 980-8575 Sendai, Japan; hiwasaki@irides.tohoku.ac.jp (H.I.); ya82@yahoo.co.jp (Y.A.); toshatto@med.tohoku.ac.jp (T.H.); 2 Emerging Infectious Diseases, Department of Internal Medicine, Graduate School of Medicine, Tohoku University, 980-8575 Sendai, Japan; chenyue69@hotmail.com (Y.C.); 009fireman@gmail.com (F.H.); tdelsi@yahoo.co.id (D.T.); hsaitoh9813135@med.tohoku.ac.jp (H.S.); 3 Adult Infectious Disease and Tropical Medicine Department, San Lazaro Hospital, 1003 Manila, Philippines; talea211md@yahoo.com; 4 National Reference Laboratory for HIV/AIDS, Hepatitis, and other STDs, STD/AIDS Cooperative Central Laboratory, 1003 Manila, Philippines; s_leano@yahoo.com (P.S.A.L.); betelan@yahoo.com (F.O.E.T.); 5 Faculty of Medicine, Universitas Padjadjaran, 40161 Bandung, Indonesia; 6 Research Institute of Pharmacological Therapeutical Development, Otsuka Pharmaceutical Co., Ltd., 771-0192 Tokushima, Japan; ohmoto.yasukazu@otsuka.jp; 7 Division of Integrated Nephrology and Telemedicine, Tohoku Medical Megabank Organization, Tohoku University, 980-8573 Sendai, Japan; kiyo@med.tohoku.ac.jp; 8 Research Center for Zoonosis Control, Hokkaido University, 001-0020 Sapporo, Japan; suzuki@czc.hokudai.ac.jp; 9 GI-CoRE Global Station for Zoonosis Control, Hokkaido University, 001-0020 Sapporo, Japan; 10 Department of Occupational Therapy, Graduate School of Health Science Studies, Kibi International University, 716-8508 Takahashi, Japan

**Keywords:** leptospirosis, defensin α1, neutrophil gelatinase-associated lipocalin (NGAL), kidney injury, *N*-acetyl-β-d-glucosidase (NAG)

## Abstract

Leptospirosis is a zoonotic disease whose severe forms are often accompanied by kidney dysfunction. In the present study, urinary markers were studied for potential prediction of disease severity. Urine samples from 135 patients with or without leptospirosis at San Lazaro Hospital, the Philippines, were analyzed. Urine levels of defensin α1 (uDA1) were compared with those of neutrophil gelatinase-associated lipocalin (uNGAL) and *N*-acetyl-β-d-glucosidase (uNAG). Serum creatinine (Cr) was used as a marker of kidney injury. The levels of uDA1/Cr, uNGAL/Cr, and uNAG/Cr were positive in 46%, 90%, and 80% of leptospirosis patients, and 69%, 70%, and 70% of non-leptospirosis patients, respectively. In leptospirosis patients, the correlation of uDA1/Cr, uNGAL/Cr and uNAG/Cr levels with serum Cr were *r* = 0.3 (*p* < 0.01), *r* = 0.29 (*p* < 0.01), and *r* = 0.02 (*p* = 0.81), respectively. uDA1/Cr levels were correlated with uNGAL/Cr levels (*r* = 0.49, *p* < 0.01) and uNAG/Cr levels (*r* = 0.47, *p* < 0.0001) in leptospirosis patients. These findings suggest that uDA1, uNGAL, and uNAG were elevated in leptospirosis patients and reflected various types of kidney damage. uDA1 and uNGAL can be used to track kidney injury in leptospirosis patients because of their correlation with the serum Cr level.

## 1. Introduction

Leptospirosis is a zoonotic disease with global distribution caused by infection with pathogenic spirochetes of the genus *Leptospira*. It is endemic in countries with humid subtropical or tropical climates and has epidemic potential [1]. Based on global data, the International Leptospirosis Society has estimated that 300,000–500,000 cases of leptospirosis occur annually, and it is assumed to be the most prevalent zoonosis [2]. Flooding and heavy rainfall have been associated with numerous leptospirosis outbreaks around the world [3], and it is also re-emerging as an infectious disease worldwide, with four cases recently identified in the Tohoku region of Japan [4].

The manifestation of leptospirosis is extremely broad, ranging from undifferentiated febrile illness to severe multisystemic disease with a high mortality rate. Weil’s syndrome, the most severe form of leptospirosis, accounts for 5%–10% of all cases and is characterized by hemorrhagic tendencies and hepatic dysfunction as well as acute renal failure [5], which develops in 16%–40% of cases and has a fatality rate of 22%–33% [5,6,7]. Additionally, delays in diagnosis and therapy are correlated with prolonged hospitalization [8]; therefore, early and accurate diagnosis of leptospirosis is essential for timely and appropriate treatment. Azithromycin and doxycycline have been reported to be effective in the treatment of this disease [9]. Azithromycin was also shown to inhibit neutrophil activation [10,11], and involvement of neutrophils and elevation of neutrophil-related activation markers such as neutrophil gelatinase-associated lipocalin (NGAL) have been reported in leptospirosis patients [12]. We recently demonstrated that urinary leptospiral DNA, some of which is presumably from phagocytized bacteria in leukocytes, increased the detection rate of leptospirosis [13]. Therefore, we tried to identify urinary markers specific to neutrophil involvement in this disease in addition to NGAL.

Compared to plasma, urine is a non-invasive source of potential biomarkers, especially for diseases linked to kidney injury [14,15]. In this study, to identify urinary markers associated with *Leptospira* infection, we performed a proteomic analysis using two urinary samples, which revealed defensin in the leptospirosis sample. Because the major source of defensin may be leukocytes, the levels of urine defensin α1 (uDA1) and other known urinary proteins such as urine NGAL (uNGAL) and urine *N*-acetyl-β-d-glucosidase (uNAG), which are markers of acute kidney injury (AKI) and tubular dysfunction, respectively, were measured in samples obtained from patients in Manila, the Philippines following the 2012 flood.

## 2. Results

### 2.1. Characteristics of the Study Population

In total, 112 confirmed leptospirosis patients, 23 non-leptospirosis patients, and eight healthy controls (HC) were enrolled in the study. The median ages of the groups were 30, 29, and 26 years old, respectively (Table 1).

### 2.2. Detection of Urinary Markers in Leptospirosis Patients

Urine samples from one leptospirosis and one non-leptospirosis patient were analyzed by 2-d gel electrophoresis. The profiles revealed >2-fold differences in the expression of certain proteins, with differences observed for >600 spots (data not shown). Five spots of interest (the highest-ranking spots in terms of differential expression) were cut from the gel and analyzed by MS, and the proteins were identified (Table 2). Among them, we focused on defensin1, the highest ranking differentially expressed protein, because it is found in leukocytes and has anti-microbial activity. Therefore, using a commercially available kit, we investigated whether uDA1 was elevated in other leptospirosis and non-leptospirosis patients.

### 2.3. Urinary DA1, NGAL, and NAG Concentrations in Leptospirosis Patients

All urinary levels of DA1, NGAL, and NAG were adjusted by uCr levels (Table 1). The levels of serum Cr were found to be significantly different among the leptospirosis, non-leptospirosis, and HC groups (*p* < 0.0001); between leptospirosis and non-leptospirosis (*p* < 0.05); and between leptospirosis and HC (*p* < 0.0001) (Table 1). The median levels of uDA1/Cr were apparently higher in leptospirosis and non-leptospirosis patients compared to HC; however, no significant differences were found among the above three groups. In contrast, significant differences in uNGAL/Cr and uNAG/Cr were found among the above three groups and between leptospirosis and HC (Table 1).

Furthermore, a receiver operating characteristic (ROC) analysis was performed to determine whether the urinary markers in this study could discriminate leptospirosis patients from non-leptospirosis patients or HC individuals. As shown in Table 3, serum Cr and uNGAL/Cr levels were able to discriminate leptospirosis patients from non-leptospirosis patients or HC individuals. However, the levels of uDA1/Cr and uNAG/Cr could not discriminate leptospirosis patients from non-leptospirosis patients, although uNAG/Cr levels were able to discriminate leptospirosis patients from non-leptospirosis and HC individuals (Table 3). According to the cutoff value based on the leptospirosis and HC groups (Table 3), uDA1/Cr was positive in 46% of leptospirosis patients, 69% of non-leptospirosis patients, and 12.5% of HC. The reason for the unexpectedly high positive rate of uDA1/Cr in non-leptospirosis patients was not clear. uNGAL/Cr was detected in 90% of leptospirosis patients, 70% of non-leptospirosis patients, and 25% of HC. uNAG/Cr was detected in 80% of leptospirosis patients and 70% of non-leptospirosis patients, but in 50% of HC. These findings indicate that all three markers were not specific to leptospirosis, but uNGAL and uNAG could be detected at higher frequencies than uDA1 in leptospirosis patients.

### 2.4. uDA1/Cr Levels Were Correlated with uNGAL/Cr and uNAG/Cr Levels in Leptospirosis Patients

**Figure 1:**
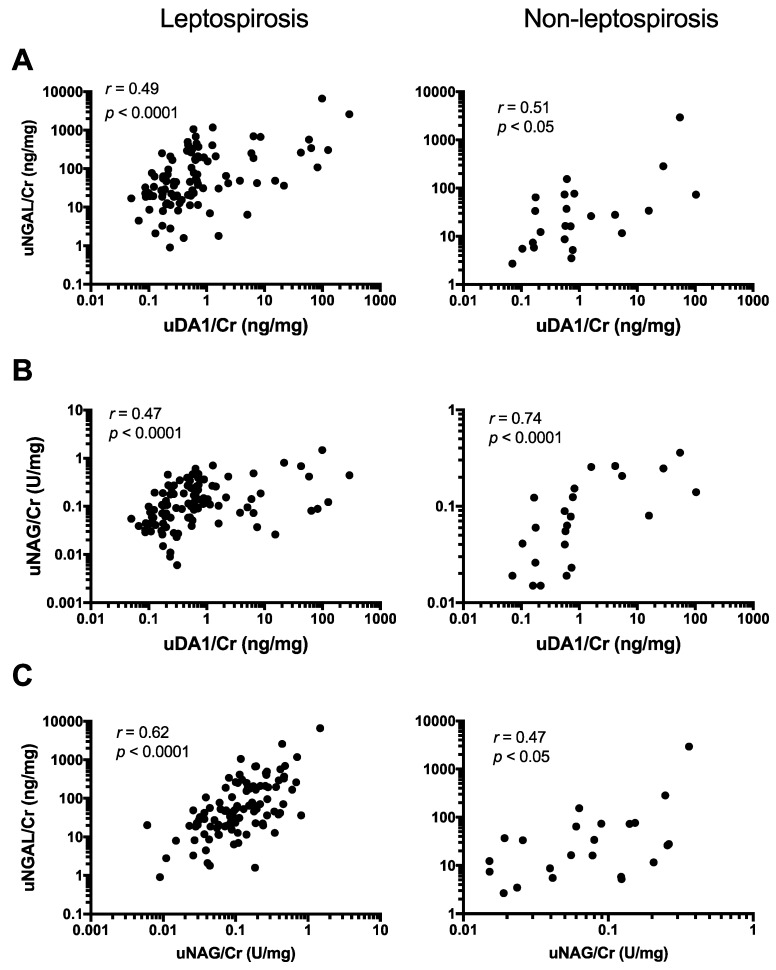
Association between urinary levels of uDA1/Cr and those of uNGAL/Cr (**A**); or uNAG/Cr (**B**); and correlation between uNGAL/Cr and uNAG/Cr levels (**C**) in leptospirosis patients and non-leptospirosis patients.

To determine the reasons for the elevation of uDA1 in leptospirosis, we investigated whether uDA1/Cr levels were associated with uNGAL/Cr and uNAG/Cr levels in leptospirosis patients. The levels of uDA1/Cr in these patients were significantly correlated with the levels of both uNGAL/Cr (*r* = 0.49; *p* < 0.0001) and uNAG/Cr (*r* = 0.47, *p* < 0.0001) (Figure 1A,B). Moreover, a significant correlation was also found between uDA1/Cr and uNGAL/Cr (*r* = 0.51, *p* < 0.05) or uNAG/Cr (*r* = 0.74, *p* < 0.0001) in non-leptospirosis patients (Figure 1A,B). In addition, a strong correlation was found between uNGAL/Cr and uNAG/Cr in both leptospirosis (*r* = 0.62, *p* < 0.0001) and non-leptospirosis patients (*r* = 0.47, *p* < 0.05) (Figure 1C). Therefore, uDA1/Cr levels were mostly correlated with uNGAL/Cr levels in leptospirosis patients, and with uNAG/Cr levels in non-leptospirosis patients.

### 2.5. Urine Levels of DA1 Are Associated with Kidney Injury

**Figure 2:**
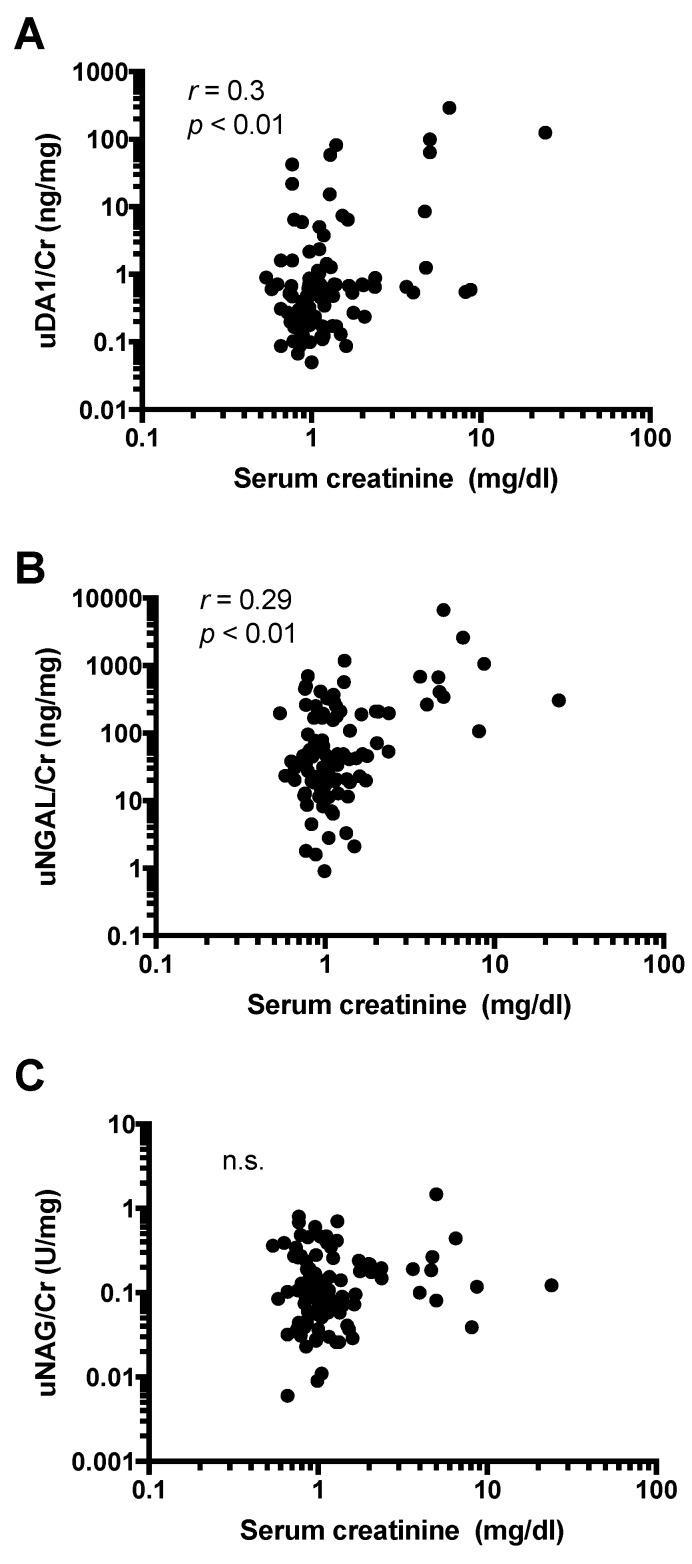
Correlation between serum Cr and urinary levels of DA1/Cr (**A**); NGAL/Cr (**B**); and NAG/Cr (**C**) in leptospirosis patients. n.s. not significant.

It was already known that uNGAL/Cr and uNAG/Cr levels are significantly correlated with serum Cr [16,17]. In this study, a positive correlation was detected between uDA1/Cr levels and levels of serum Cr, which is a marker for kidney injury, among leptospirosis patients (*r* = 0.3; *p* < 0.01) (Figure 2A). In addition, uNGAL/Cr levels were significantly associated with serum Cr levels (*r* = 0.29, *p* < 0.01) (Figure 2B); however, no correlation was found with uNAG/Cr levels in leptospirosis patients (*r* = 0.02, *p* = 0.81) (Figure 2C). These results suggest the possible involvement of uDA1/Cr and uNGAL/Cr in kidney injury. None of these correlations were found in non-leptospirosis patients.

**Figure 3:**
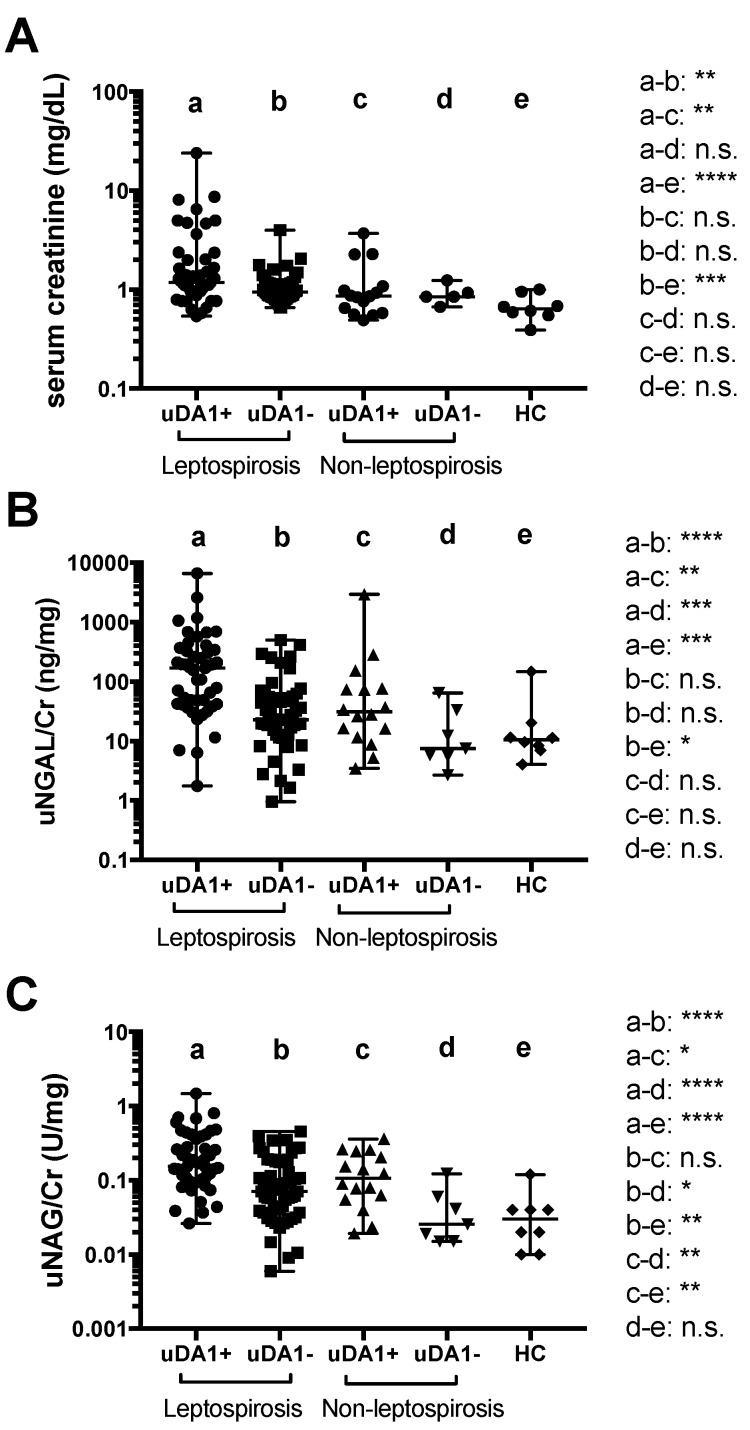
Differences in serum Cr (**A**); uNGAL/Cr (**B**); and uNAG/Cr (**C**) levels between uDA1-positive and -negative groups in leptospirosis and non-leptospirosis patients and HCs. * *p* < 0.05; ** *p* < 0.01, *** *p* < 0.001, **** *p* < 0.0001, n.s. not significant.

We also compared serum Cr, uNGAL/Cr, and uNAG/Cr levels in uDA1-positive and -negative groups (cut-off 0.54, Table 3) to determine whether uDA1 in leptospirosis reflects the severity of kidney injury. The levels of serum Cr and uNGAL/Cr were higher in uDA1-positive groups than in uDA1-negative groups only in leptospirosis patients (Figure 3A,B). Moreover, uNAG/Cr levels were significantly higher in uDA1-positive groups than in uDA1-negative groups in both leptospirosis patients and non-leptospirosis patients (Figure 3C).

### 2.6. Association between Urinary Markers and Dipstick Parameters

**Figure 4:**
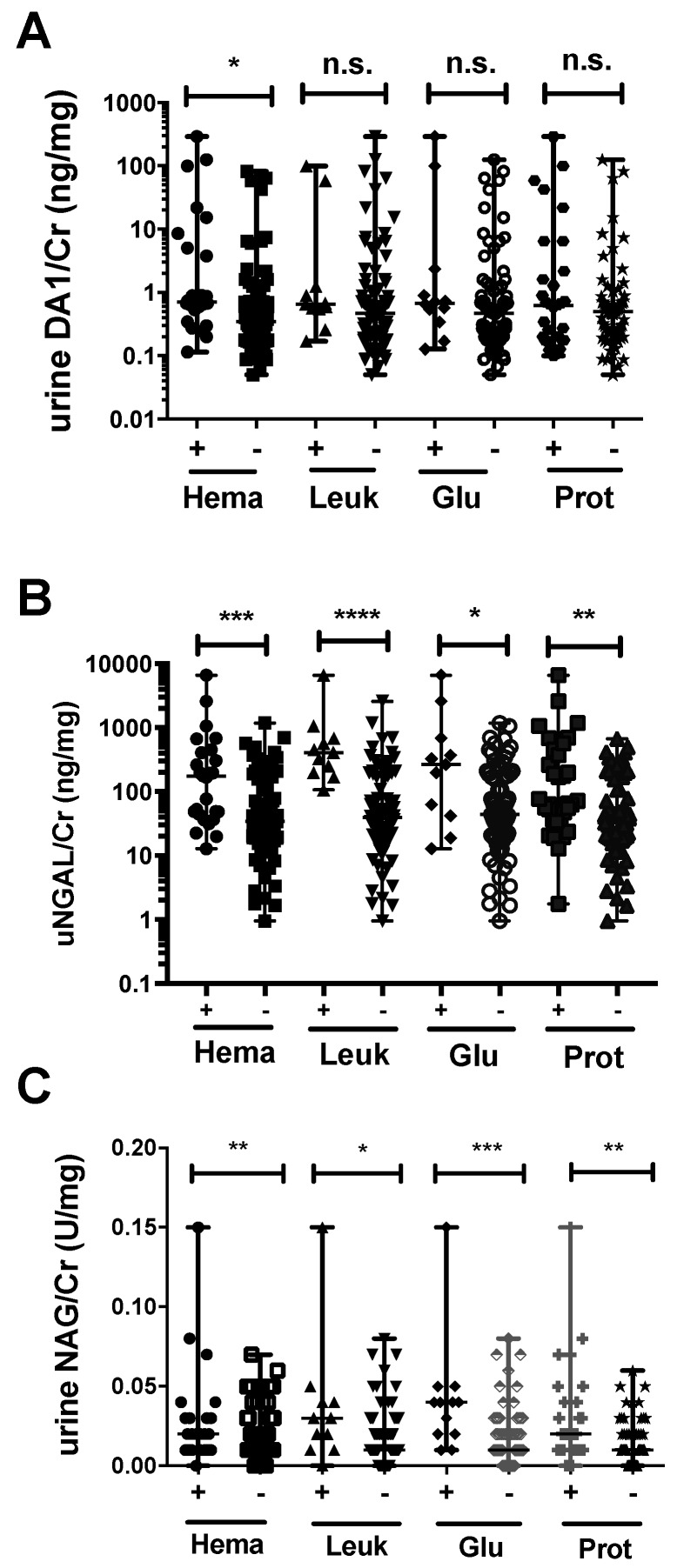
Urinary levels of DA1 (**A**); NGAL (**B**); and NAG (**C**) in hematuria (Hema)-, leukocyte (Leuk)-, glucose (Glu)-, and proteinuria (Prot)-positive or -negative groups among leptospirosis patients. * *p* < 0.05, ** *p* < 0.01, *** *p* < 0.001, **** *p* < 0.0001, n.s. not significant.

Further associations between the above-described urinary markers and urinary dipstick parameters were analyzed. uDA1/Cr levels were significantly higher in the hematuria-positive group only (*p* < 0.05) (Figure 4A), whereas uNGAL/Cr and uNAG/Cr levels were significantly higher in the hematuria-, leukocyte-, glucose-, and proteinuria-positive groups compared to the corresponding negative groups in leptospirosis patients (Figure 4B,C). In non-leptospirosis patients, uDA1/Cr and uNGAL/Cr levels were significantly higher in the hematuria-positive group only (data not shown).

## 3. Discussion

The results presented here reveal for the first time the presence of DA1 in the urine of leptospirosis patients. We demonstrated a significant correlation of uDA1/Cr levels with uNGAL/Cr, uNAG/Cr, and serum Cr levels in leptospirosis patients. Furthermore, serum Cr, uNGAL/Cr, and uNAG/Cr levels were significantly higher in the uDA1-positive than in the negative group, indicating that uDA1 levels reflect the severity of kidney injury in leptospirosis. Increased uDA1 levels were observed in 46% of leptospirosis patients but also in 69% of non-leptospirosis patients. The reason for unexpectedly high positive rate of uDA1/Cr in non-leptospirosis patients is not clear. The uDA1/Cr level was correlated with the level of uNGAL/Cr, which is a 25 kDa member of the lipocalin protein family that was first identified in activated human neutrophils [18], and also with the levels of uNAG/Cr, a lysosomal enzyme abundant in proximal kidney tubule cells. An increase of uNAG levels and tubular dysfunction in leptospirosis patients was reported previously [19], and serum and urine NGAL has been shown to be associated with acute kidney failure [12,20]. However, it has also been reported that urinary tract infections such as cystitis are also associated with uNGAL levels in the absence of AKI, as neutrophils secrete NGAL [21,22]. These results may indicate the involvement of neutrophils in *Leptospira* infection of the kidney.

Neutrophil infiltration in the kidney has been observed in biopsy specimens of AKI patients [23]. In leptospirosis patients with kidney dysfunction, interstitial nephritis is the major finding in biopsies and is accompanied by significant neutrophil and monocyte infiltration [5,24]. Neutrophils play an important role in the phagocytosis of *Leptospira* if the pathogen is opsonized by a specific IgG [25], and *L. icterohemorrhagiae* infection and *Leptospira* peptidoglycans induce endothelial cells adhesiveness for polynuclear leukocytes [26]. Thus, *Leptospira* infection of the kidney leads to the recruitment of neutrophils that release antibacterial proteins such as defensin and NGAL.

Defensins (also known as human neutrophil peptides) are small cationic peptides (3–4 kDa) with broad antibacterial, antiviral, and antifungal activities [27]. Human α and β defensins differ in terms of their disulfide bond patterns, and the former are predominantly found in neutrophils and in small intestinal Paneth cells [28]. An in vitro study has shown that defensins have anti-*Leptospira* activity [29]. DA1 in synovial fluid provides higher sensitivity and specificity than other markers for identifying periprosthetic joint infection in patients [30]; in our study, 46% of leptospirosis patients had elevated levels of uDA1/Cr, which were significantly associated with the levels of uNGAL/Cr. In ischemic or nephrotoxic renal injury, NGAL is abundantly expressed in the kidneys and released into the urine. NGAL levels increase within 2 h of renal injury, making it an early and sensitive biomarker [31,32,33]. In the present study, uNGAL/Cr was positive in 90% of leptospirosis patients, which was higher than the positive rate for uDA1/Cr. This is likely because uNGAL is also released from proximal tubule cells or α-intercalated cells, in addition to neutrophils [21,34]. Moreover, patients who were positive for uDA1/Cr exhibited higher levels of serum Cr than those who were negative, suggesting that uDA1 levels reflect kidney injury status in leptospirosis.

NAG has a relatively large molecular weight of 130–140 kDa, which prevents its passage through the glomerular basal membrane [35]. Nonetheless, it is a sensitive marker for kidney damage and was detected at a high positive rate in 80% of leptospirosis patients in this study, suggesting that proximal tubular damage was present in these individuals. The levels of uNAG/Cr and uNGAL/Cr were significantly higher in the hematuria-, leukocyte-, glucose-, and proteinuria-positive groups compared to the corresponding negative groups in leptospirosis patients, indicating that uNAG/Cr and uNGAL/Cr levels could reflect kidney damage also detectable by urinary dipstick test. Furthermore, uNGAL and uNAG as well as uDA1 were also found in non-leptospirosis patients, indicating that all three markers were not specific to leptospirosis; additionally, uNGAL and uNAG were detected at higher frequencies than uDA1 in leptospirosis patients. We therefore suggest that the urinary markers examined here reflect different types of damage caused by kidney failure resulting from *Leptospira* infection; uDA1 may reflect the involvement of neutrophils during kidney injury, whereas uNGAL and uNAG reflect both neutrophil involvement and tubular dysfunction. Our previous data showed the presence of *Leptospira* DNA in the urine of some of the patients [13], and uDA1 was also positive in kidney derived-DNA-positive cases (unpublished observation), further supporting that neutrophil activation is caused by bacteria. Very recently, it was shown that the leptospiral outer membrane protein LipL32 induces inflammation, including leukocyte infiltration and kidney injury, in zebrafish larvae [36]. Therefore, therapies against neutrophil-derived inflammation would be recommended in uDA1-positive leptospirosis, and in fact azithromycin, which is known to inhibit neutrophil activation and also to have antibacterial activity [11], was demonstrated to be effective against this disease [9].

There are several limitations in our study. One limitation was that spot urine samples were used; typically, urinary analysis over a 24-hour period is necessary for comparisons and assessment of the utility of markers in clinical practice. Additionally, clinical data for estimated glomerular filtration rate (eGFR), diabetes, hypertension, heart failure, underlying chronic kidney disease, C-reactive protein (CRP), mean arterial pressure, and hemoglobin for these patients were not collected in this study. Furthermore, this study was performed in a single institution, a very low number of control subjects was enrolled, and only two samples were used for the proteomics analysis. Consequently, the results may not be directly applicable to other patient populations. In addition, clarifying the exact role of uDA1 in leptospirosis requires a large-scale longitudinal study that includes clinical data on kidney function. Finally, this is a retrospective study, which limits the generalization of its findings. Nonetheless, our findings provide the first identification and quantitation of uDA1 levels in leptospirosis patients, and suggest that uDA1 can serve as a biomarker to track kidney injury.

## 4. Materials and Methods

### 4.1. Study Subjects

A retrospective study was performed in 135 patients admitted to San Lazaro Hospital (Manila, Philippines) in 2012 with clinical suspicion of leptospirosis; 112 of them were confirmed positive with one of following diagnostic tools using serum or urine samples: the microscopic agglutination test [37], immunochromatographic assay (Standard Diagnostics, Yongin, Korea), enzyme-linked immunosorbent assay (ELISA; Diagnostic Automation, Calabasas, CA, USA), loop-mediated isothermal amplification, and real-time PCR [13,38]. The remaining 23 patients were confirmed to have non-leptospirosis illnesses of unknown origin and served as controls. Blood/spot urine samples were obtained from each patient and serum/urine supernatants were collected by centrifugation at 3000 rpm for 10 min. Sample aliquots were stored at −80 °C until use.

### 4.2. Ethics Statement

The study was conducted in accordance with the Declaration of Helsinki and was approved by the Ethics Committee of San Lazaro Hospital (No. 2541249-01-06-15, approved 7 January 2015) (Manila, Philippines) and Tohoku University Hospital (No. 2013-1-224, approved 19 August 2013) (Sendai, Japan). The data were analyzed anonymously and no consent was obtained. The ethics committees waived the need for obtaining consent.

### 4.3. Two-Dimensional Electrophoresis (2-DE) and Mass Spectrometry (MS) Analysis

To detect candidate marker proteins for leptospirosis, a proteomics analysis using 2-DE and MS was performed as previously described [39] using urine samples from leptospirosis (No. 13) and non-leptospirosis (No. 62) patients. Urine samples were treated with the 2D Clean-up kit (Bio-Rad, Hercules, CA, USA) and dissolved in rehydration buffer. Protein concentration was measured by the Bradford method. 2-DE was performed in an electrophoresis system by isoelectric focusing using an IPG Ready Strip gel (Bio-Rad) in the first dimension and by sodium dodecyl sulfate-polyacrylamide gel electrophoresis with a 10%–16% gradient gel in the second dimension using 110 μg of sample. Images were acquired by scanning SYPRO Ruby-stained gels (Life Technologies, Carlsbad, CA, USA) using Molecular Imager FX (Bio-Rad). Spot patterns on the gels for patients No. 13 and 62 were compared using Progenesis Same Spots (Nonlinear Dynamics, Durham, NC, USA). Spots of interest were selected and trypsin-treated peptides were analyzed by matrix-assisted laser desorption/ionization or liquid chromatography–tandem MS. Data were searched in the NCBInr protein database for identification.

### 4.4. Measurement of Urinary Markers and Creatinine

uDA1 was measured using the human DA1 ELISA kit (Cosmo Bio Co., Wuhan, China) according to the manufacturer’s instructions. uNGAL levels were measured by ELISA as previously described [40], and uNAG was measured at the Special Reference Laboratory (SRL) (Hachioji, Japan). Values for uDA1, NGAL, and NAG are expressed as ng/mL, ng/mL, and U/L, respectively. Urinary marker levels were determined relative to the levels of urinary Cr (uCr), which were determined using the Cr parameter assay kit (R&D Systems, Minneapolis, MN, USA) according to the manufacturer’s instructions. Serum Cr levels were measured at the SRL.

### 4.5. Dipstick Analysis

A dipstick kit (Eiken Chemical Co., Tokyo, Japan) was used to determine the presence of red blood cells, leukocytes, albumin, and glucose in urine.

### 4.6. Statistical Analysis

Data are expressed as the median and range. Statistical differences between two groups were assessed by the Mann–Whitney test. Correlations between two groups were analyzed using the Spearman rank correlation test. The differences in uDA1/Cr, uNGAL/Cr, uNAG/Cr, and serum Cr levels among the leptospirosis, non-leptospirosis, and HC groups were assessed using the Kruskal–Wallis test, and further differences between the two groups were measured using Dunn’s multiple comparison test. These analyses were performed using GraphPad Prism 6 software (GraphPad, San Diego, CA, USA). Furthermore, ROC analysis was performed to study the predictive markers for distinguishing leptospirosis patients from non-leptospirosis patients or HC using Medcalc statistical software version 16.8 (Ostend, Belgium). A significant difference was assumed at *p* < 0.05.

## 5. Conclusions

Our findings provide the first identification and quantitation of DA1 levels in urine of leptospirosis patients. It was found that uDA1, uNGAL, and uNAG were elevated in leptospirosis patients and reflected various types of kidney damage. In addition, uDA1 and uNGAL can be used to track kidney injury in leptospirosis.

## Figures and Tables

**Table 1:** Basic and clinical characteristics of the study groups.

Variables	Leptospirosis	Non-Leptospirosis	HC
Demographic data			
Sex: male (n (%))	94 (94.9)	19 (82.6)	2 (25.0)
Age in years (median (range))	30 (12–67)	29 (19–51)	26 (22–43)
Markers (median (range))			
uDA1/Cr	0.498 (0.05–292.03)	0.614 (0.07–103.35)	0.22 (0.088–0.995)
uNGAL/Cr **^a,c^**	46.38 (0.944–6662.4)	26.23 (2.67–2941.5)	10.45 (4.04–147.19)
uNAG/Cr **^a,c^**	0.107 (0.006–1.469)	0.077 (0.0151–0.36)	0.03 (0.008–0.116)
Serum Cr **^a,b,c^**	1.01 (0.54–24.04)	0.855 (0.49–3.71)	0.69 (0.39–1)

Cr, creatinine; HC, healthy control; uDA1, urinary defensin α1; uNAG, urinary *N*-acetyl-β-d-glucosidase; uNGAL, urinary neutrophil gelatinase-associated lipocalin; **^a^** significant differences among leptospirosis, non-leptospirosis, and HC groups; **^b^** significant differences between leptospirosis and non-leptospirosis groups; **^c^** significant differences between leptospirosis and HC groups.

**Table 2:** Results of mass spectrometry analysis of top five differentially expressed spots.

**Spot Sample ID**	2056	2060	1648	1804	1293
**Fold Differential (A > B)**	55.3	45.7	41.7	40	25
**Proteins Matched with Database**	neutrophil defensin 1 preproprotein	neutrophil defensin 1 preproprotein	Retinol binding protein 4, plasma	pancreatic stone protein	hemopexin, isoform CRA_a
			agrin precursor, partial	lipocalin-1 isoform 1 precursor	
			regenerating protein (reg)	prolactin-inducible protein precursor	
				neudesin precursor	
				protein AMBP preproprotein	
				immunoglobulin lambda light chain, partial	
				glia maturation factor gamma	
				SMT3A protein	
				semenogelin	
				DskA-type zinc finger protein (organism: *Shewanella piezotolerans WP3*)	
				phosphatidylglycerophosphate synthase-like protein (organism: *Trypanosoma brucei* brucei strain 927/4 GUT at 10.1)	

**Table 3:** Summary of ROC curve analysis.

	Lepto & Non-Lepto				Lepto & Non-lepto, HC				Lepto & HC			
ROC analysis	uDA1/Cr	uNGAL/Cr	uNAG/Cr	Ser Cr	uDA1/Cr	uNGAL/Cr	uNAG/Cr	Ser Cr	uDA1/Cr	uNGAL/Cr	uNAG/Cr	Ser Cr
AUC	0.568	0.645	0.621	0.67	0.505	0.686	0.679	0.743	0.676	0.803	0.848	0.89
*p*-value	0.34	0.03	0.06	0.02	0.94	0.0008	0.001	<0.0001	0.06	0.0001	<0.0001	<0.0001
Youden index	0.245	0.292	0.212	0.344	0.11	0.362	0.292	0.4	0.35	0.623	0.65	0.74
Cut-off	0.55	16.43	0.08	0.93	0.53	16.43	0.08	0.68	0.54	11.43	0.04	0.68
Sensitivity (%)	54.9	81.37	64.7	64.36	52.94	81.37	64.71	94.06	47.06	87.25	77.45	94.06
Specificity (%)	69.6	47.83	56.5	70	58.06	54.84	64.52	46.67	87.5	75	87.5	80

Lepto, leptospirosis; HC, healthy control; Cr, creatinine; uDA1, urinary defensin α1; uNAG, urinary *N*-acetyl-β-d-glucosidase; uNGAL, urinary neutrophil gelatinase-associated lipocalin; Ser, serum.
